# Telephone Survey to Assess Influenza-like Illness, United States, 2006

**DOI:** 10.3201/eid1401.070265

**Published:** 2008-01

**Authors:** Joseph L. Malone, Mohammad Madjid, S. Ward Casscells

**Affiliations:** * University of Texas Health Science Center, Houston, Texas, USA; † Texas Heart Institute, Houston, Texas, USA; ‡ St. Luke’s Episcopal Hospital, Houston, Texas, USA

**Keywords:** Disaster planning, disease outbreaks/prevention and control, influenza, human/diagnosis/prevention and control, population surveillance/methods, telephone, research

## Abstract

Although current national response plans assume that most influenza-infected patients would stay home during a pandemic, surveillance systems might be overwhelmed and unable to monitor their health status. We explored the feasibility of using a nationwide telephone survey to monitor at-home patients. Of randomly selected adults surveyed during low influenza activity months (April–October 2006, surveillance weeks 17–41), 86% (7,268/8,449) agreed to answer questions about health status and influenza-like illness symptoms. Three percent (230/7,628) self-reported “flu.” A subset (0.9%, 68/230) self-reported fever. In comparison, the Centers for Disease Control and Prevention’s Sentinel Provider Network reported clinical influenza-like illness rates of 1.2%, 0.9%, and 1.2% for weeks 17, 20, and 41, respectively. The consistency between information obtained by telephone and surveillance data warrants further studies to determine whether telephone surveys can accurately monitor health status during seasonal influenza peaks and to augment current surveillance systems during a pandemic.

## Abstract 2

This method offers a potentially feasible means to monitor patients at home**.**

The US Department of Health and Human Services (DHHS), Centers for Disease Control and Prevention (CDC), and other government agencies have been working to strengthen public health systems and improve strategies for monitoring and controlling pandemic influenza. According to the US Homeland Security Council’s National Strategy for Pandemic Influenza Implementation Plan, “... the public health community must have situational awareness of the evolution of disease that can only come from connectivity to the emergency departments and other acute care settings where patients with influenza are presenting. The interpandemic period presents an opportunity to establish and test these relationships” (*1*).

CDC currently supports the influenza surveillance reporting systems listed in Table 1 (*2**,**3*). Surveillance goals of the DHHS pandemic influenza plan are to 1) provide an early-warning system; 2) detect increases in influenza-like illness (ILI) at the local level; 3) monitor the impact of influenza on health (e.g., track outpatient visits, hospitalizations, and deaths); 4) track trends in influenza disease activity; and 5) identify severely affected populations (*3*). Outpatient surveillance of ILI is emphasized at the national, regional, and state levels through CDC’s Sentinel Provider Network (SPN), which gathers and summarizes influenza illness surveillance reports from ≈2,300 healthcare providers nationwide (*2*). CDC plans to analyze daily reports of ambulatory patients with influenza that are accessed through the BioSense surveillance system (*4**,**5*) and use existing emergency department symptom-monitoring systems (*2*). This approach will strengthen systems that support situational awareness of influenza outbreaks, support geographic completeness and frequency of reporting, and ensure sustainable collection of ILI data during a pandemic.

**Table 1:** Current influenza surveillance systems reporting to the US Centers for Disease Control and Prevention*

System	Description	Reporting frequency
Sentinel Provider Network (SPN)	Reports percentage of outpatient visits for influenza-like illness and total patients seen for any reason from a network of 2,300 nationwide healthcare providers	Weekly
Emerging Infections Program (EIP)	Reports laboratory-confirmed influenza-associated hospitalizations of children <18 years of age in 11 US communities	Biweekly
New Vaccine Surveillance Network (NVSN)	Reports laboratory-confirmed influenza-associated hospitalizations of children <5 years of age in 3 US communities	Biweekly
122 Cities Mortality Reporting System	Reports pneumonia- and influenza-related deaths and total no. deaths in 122 US cities	Weekly
National Notifiable Disease Surveillance System (NNDSS)	Reports influenza-associated pediatric deaths recorded by participating state health departments	Weekly
State and Territorial Epidemiologists Report	Reports overall level of influenza activity in respective states, territories, or both	Weekly
US World Health Organization and Global Influenza Surveillance Network; National Respiratory and Enteric Virus Surveillance System (NREVSS)	Report no. influenza laboratory tests performed and no. positive results by type and, in some cases, subtype	Weekly

*See (*2*,*3*).

The DHHS Pandemic Influenza Plan notes that “most patients with pandemic influenza will be able to remain at home during the course of their illness and can be cared for by other family members or others who live in the household” (*6*). Local health districts, although responsible for monitoring the care of these at-home patients and maintaining continuity of services, may be too overwhelmed to do so. Because of the expected high demand for medical services during a pandemic influenza emergency, SPN providers may also be overwhelmed, may have difficulty finding time to report outpatient ILI status in a timely manner, and may be unable to monitor ILI patients at home (*4*). Inadequate surveillance will result in reduced situational awareness at local, state, and federal levels. Currently, none of the functioning federal public health systems has plans in place to identify or monitor the health status of at-home patients with ILI symptoms.

We investigated whether a national telephone survey system would be feasible to fill the gap in surveillance. To determine whether national and local situational awareness of influenza could be improved by monitoring at-home patients in a systematic way, we partnered with a public opinion research company to administer and analyze the results of a telephone survey. We report the results of that survey.

## Methods

Using national pandemic influenza planning documents, CDC guidance, and the recent medical literature, we developed a telephone survey questionnaire (Table 2) that asked “Do you have the flu?” and questions about symptoms such as fever, cough, and sore throat. This design allows comparison of the survey data with the SPN’s definition of ILI (i.e., fever [>100°F (37.8°C)] and cough and/or sore throat in the absence of a known cause other than influenza). Possible risk factors for exposure to novel avian influenza strains through live poultry (*7*), foreign travel (*8**,**9*), and cats (*10*) were also addressed in the survey.

**Table 2:** Telephone survey questions*

A. Do you have a cat(s)?
A.1. How many?
A.2. How many are recently sick?
A.3. How many have died?
B. Do you have chickens?
B.1. What proportion, if any, are sick?
B.2. Have there been more deaths of chickens than usual this week?
C. Now we would like to ask you a few questions related to a research study conducted by the University of Texas Science Center at Houston. Are you willing to participate?
C.1. Do you have the flu?
C.1.1. Have you received your flu shot this year?
C.1.2. What is your body temperature (fever)?
C.1.2. Do any of your contacts have flu?
C.1.3. Do you have shaking chills?
C.1.4. Do you have body aches and muscle pain, in the back, arms or legs?
C.1.5. Do you have a cough?
C.1.6. Do you have a runny nose?
C.1.7. Are you short-winded?
C.1.8. Do you have a sore throat?
C.1.9. Have you traveled to Southeast Asia recently?
C.1.10. How long have you had these symptoms?

*Questions A–C were asked of all adults taking the telephone survey. Only respondents who answered “yes” to Question C were asked Question C.1; only respondents who answered “yes” to C.1 were asked the follow-up questions C.1.1 through C.1.10.

To explore the feasibility of using this telephone survey, we partnered with Zogby International (Utica, NY, USA) (*11*), a private public opinion research company experienced at collecting extensive demographic and socioeconomic information through nationwide telephone surveys. A protocol to perform telephone surveys was submitted January 11, 2006 for institutional review board (IRB) review, subsequent questionnaire modifications were approved March 20, 2006, and the first survey was completed the week of April 24, 2006, ≈110 days later. Monthly, ≈1,205 randomly selected adults, who were already providing demographic and socioeconomic information for other Zogby International telephone surveys, were asked to participate in a research study about influenza. Participants answered follow-up questions over 5-day intervals ending on each of the following dates in 2006 (corresponding to CDC influenza surveillance calendar weeks) (*12*): April 24 (week 17); May 16 (week 20); June 6 (week 23); July 25 (week 30); August 15 (week 33); September 14 (week 37); and October 12 (week 41). These dates were selected as a convenience sample based upon having received necessary IRB approvals and having the availability of telephone survey capacity by our telephone surveying partners. Telephone numbers for the surveys were randomly selected from commercially available national data sets of residential directory-listed telephone numbers. The probability of selection for the telephone survey was adjusted to be proportional to population sizes within area codes and telephone exchanges. As many as 6 calls were made to reach a sampled phone number, and the procedures did not result in the same telephone numbers selected to be surveyed for each subsequent month.

One adult from each randomly sampled household was asked to answer the survey questionnaire by telephone. All interviews were conducted by Zogby International’s general interviewers, who are monitored by supervisors to assess adherence to surveying standards. For this study, the interviewer-to-supervisor ratio was 12:1. Quality control checks of interviewer performance were conducted on 10% of all calls. The survey was performed according to a protocol created by medical researchers at the University of Texas Health Science Center at Houston and approved by the university’s Institutional Review Board. Survey results were tabulated, analyzed, and correlated by the study authors with nationwide influenza surveillance reports from CDC (Table 1) (*2*) for the 2005–06 and 2006–07 influenza seasons (*1**,**2**,**12*).

## Results

Of 8,449 adults contacted, 7,268 (86%) agreed to participate in the survey. Participants were from representative socioeconomic and racial/ethnic groups (Table 3) across the United States. The overall monthly participation rate was 83%–87%. Participation rates for men and women were similar.

**Table 3:** Results of a national telephone survey of US adults at home (2006) regarding influenza-like illness, cats, and live chickens in the household*

Information and questions	Survey date						
	24 Apr 2006 (wk 17)	16 May 2006 (wk 20)	06 Jun 2006 (wk 23)	25 Jul 2006 (wk 30)	15 Aug 2006 (wk 33)	14 Sep 2006 (wk 37)	12 Oct 2006 (wk 41)
No. participants/no. surveyed (%)	1,039/1,209 (86)	1,036/1,200 (86)	1,068/1,205 (89)	1,031/1,200 (86)	1,048/1,214 (86)	1,001/1,210 (83)	1,045/1,211 (86)
No. participants who answered “yes” to the following (%)							
“Do you have the flu?”	41 (4)	32 (3)	30 (3)	46 (5)	23 (2)	23 (2)	35 (3)
“Do you have any cats?”	379 (37)	316 (30)	298 (28)	331 (32)	354 (34)	330 (33)	330 (32)
“Do you have any chickens?”	21 (2)	15 (1)	39 (4)	44 (4)	18 (2)	14 (1)	33 (3)
Sex, no. participants/no. surveyed (%)							
Male	514/583 (88)	491/578 (85)	509/581 (88)	492/578 (85)	509/585 (87)	492/583 (84)	520/584 (89)
Female	525/626 (84)	545/662 (82)	558/624 (90)	539/622 (87)	539/629 (86)	509/627 (81)	526/627 (84)
Age, y, no. participants/no. surveyed (%)							
18–29	195/239 (82)	186/237 (78)	198/237 (83)	188/236 (80)	192/236 (81)	207/236 (88)	211/237 (89)
30–49	418/477 (88)	425/475 (90)	438/474 (92)	416/472 (88)	422/472 (89)	395/472 (84)	416/474 (88)
50–64	248/274 (90)	247/273 (90)	240/273 (88)	250/271 (92)	244/272 (90)	227/272 (84)	242/273 (89)
>65	165/203 (82)	169/202 (84)	176/200 (88)	165/201 (82)	164/201 (82)	153/201 (76)	153/201 (76)
Ethnicity, no. participants/no. surveyed (%)							
White	787/896 (88)	763/878 (87)	795/887 (89)	765/879 (87)	775/884 (88)	733/884 (83)	769/885 (87)
Hispanic	96/119 (81)	90/119 (76)	107/120 (89)	93/119 (78)	104/120 (87)	105/119 (88)	101/120 (84)
African American	106/131 (81)	117/131 (89)	115/132 (87)	121/131 (92)	103/131 (79)	100/131 (76)	116/132 (88)
Asian	19/24 (79)	21/24 (88)	23/24 (96)	20/24 (83)	18/24 (75)	19/24 (79)	20/24 (83)
Other	19/24 (79)	33/36 (92)	24/36 (67)	24/36 (67)	33/36 (92)	30/36 (83)	27/36 (75)
Marital status and children, no. participants/no. surveyed (%)							
Married	594/676 (88)	556/654 (85)	582/651 (89)	576/644 (89)	567/656 (86)	597/688 (87)	606/702 (86)
Children in home	336/385 (87)	334/385 (87)	406/442 (92)	351/393 (89)	343/389 (88)	NA	NA
Home locale, no. participants/no. surveyed (%)							
Large city	259/292 (89)	285/322 (89)	286/326 (88)	262/313 (84)	267/292 (91)	273/332 (82)	296/341 (87)
Small city	270/342 (79)	300/355 (85)	265/317 (84)	281/343 (82)	280/332 (84)	310/382 (81)	282/327 (86)
Suburb	230/259 (89)	161/186 (87)	223/231 (97)	211/235 (90)	171/203 (84)	182/213 (85)	205/228 (90)
Rural	270/312 (87)	279/325 (86)	283/319 (89)	262/290 (90)	327/380 (86)	232/275 (84)	257/302 (85)
Region, no. participants/no. surveyed (%)							
East	244/278 (88)	242/276 (88)	250/277 (90)	239/276 (87)	243/279 (87)	234/278 (84)	239/279 (86)
South	263/314 (84)	278/312 (89)	262/313 (84)	274/312 (88)	266/316 (84)	284/315 (90)	276/315 (88)
Central	328/375 (88)	324/372 (87)	325/374 (87)	324/372 (87)	318/376 (85)	277/375 (74)	323/375 (86)
West	204/242 (84)	192/240 (80)	231/241 (96)	194/240 (81)	220/243 (91)	206/242 (85)	207/242 (86)
Education, no. participants/no. surveyed (%)							
Less than high school	345/52 (87)	213/240 (89)	178/240 (74)	191/240 (80)	210/242 (87)	180/241 (75)	205/242 (85)
High school graduate	221/265 (84)	266/335 (79)	304/336 (90)	279/336 (83)	285/339 (84)	273/338 (81)	297/339 (88)
Some college	306/356 (86)	250/276 (91)	259/276 (94)	242/276 (88)	236/278 (85)	238/278 (86)	241/278 (87)
More than college	466/534 (87)	305/347 (88)	323/348 (93)	318/348 (91)	314/351 (90)	308/350 (88)	300/351 (86)

*Data regarding annual income available from authors upon request. NA, not available.

Of the 7,268 adults surveyed by telephone during 7 surveys (CDC weeks 17–41), 2,337 (32%) said that they had cats in the home, 184 (2.5%) lived in close contact with chickens, and 230(3.2%) answered “yes” when asked “Do you have the flu?” Of the 230 adults who answered yes to having the flu, only 49 (21%) reported having received an annual influenza vaccine, 68 (30%) reported having fever or abnormal body temperatures, 93 (40%) reported having a cough, and 89 (39%) reported having a sore throat (Table 4). According to CDC influenza surveillance reports for weeks 17, 20, and 41 (Table 5), influenza activity nationwide peaked in early March 2006 (at approximately week 10); influenza B virus was the most commonly isolated influenza virus during weeks 17 and 20, and influenza A virus was the most frequently isolated virus in week 41 (*12*). Data for weeks 21–39 in 2006 were not reported by CDC in the 2005–06 and 2006–07 influenza seasons (*12*).

**Table 4:** Health status information for patients answering “yes” to “Do you have the flu?” and possible risk factors for pandemic influenza illness, United States, 2006

Information and questions	Survey date						
	24 Apr 2006 (wk 17)	16 May 2006 (wk 20)	06 Jun 2006 (wk 23)	25 Jul 2006 (wk 30)	15 Aug 2006 (wk 33)	14 Sep 2006 (wk 37)	12 Oct 2006 (wk 41)
No. participants in survey	1,039	1,036	1,068	1,031	1,048	1,001	1,045
No. who answered “yes” to “Do you have the flu?”	41	32	30	46	23	23	35
If answered “yes” to “Do you have the flu?” self-responded “yes” to the following, no. (%)							
Annual influenza vaccination	12 (29)	14 (44)	14 (47)	30 (65)	5 (22)	2 (9)	2 (6)
Elevated body temperature	11 (27)	22 (69)	9 (30)	7 (15)	1 (4)	13 (57)	5 (14)
Contact with others having flu	16 (39)	6 (19)	14 (47)	24 (52)	13 (57)	8 (35)	3 (9)
Cough	15 (37)	12 (38)	16 (53)	24 (52)	5 (22)	10 (44)	11 (31)
Aches	23 (56)	25 (78)	9 (30)	32 (70)	9 (39)	16 (70)	7 (20)
Chills	9 (22)	3 (9)	4 (13)	21 (46)	9 (39)	10 (43)	9 (26)
Runny nose	22 (54)	23 (72)	8 (27)	23 (50)	5 (22)	14 (61)	9 (26)
Short-windedness	9 (22)	7 (22)	8 (27)	29 (63)	12 (52)	9 (39)	2 (6)
Sore throat	15 (37)	22 (69)	5 (17)	23 (49)	8 (35)	6 (26)	10 (29)
Southeast Asia travel	0 (0)	1 (3)	0 (0)	19 (41)	2 (9)	1 (4)	1 (3)
Illness duration <10 days	24 (59)	21 (66)	16 (53)	26 (57)	7 (30)	18 (78)	20 (57)
Table 5. Summary of CDC national influenza surveillance reporting, United States, 2006*							

Influenza surveillance system	Data reported	Survey date†		
		29 Apr 2006 (wk 17)	20 May 2006 (wk 20)	14 Oct 2006 (week 41)
Sentinel Provider Network (SPN)	Outpatient visits	ILI rate = 1.2% (<2.2% national baseline)	ILI rate = 0.9% (<2.2% national baseline)	ILI rate =1.2% (<2.1% national baseline)
Emerging Infections Program (EIP)	Laboratory-confirmed influenza hospitalizations of children <18 years of age	1.08/10,000 (cumulative)	1.21/10,000	Not reported
New Vaccine Surveillance Network (NVSN)	Laboratory-confirmed influenza-associated hospitalizations of children <5 years of age	4.3/10,000 (cumulative)	5.4/10,000	Not reported
122 Cities Mortality Reporting System	Pneumonia and influenza deaths compared with all causes of death	7.1% < threshold 7.8%	6.3% < threshold 7.4%	5.6% < threshold 6.38%
National Notifiable Disease Surveillance System	Seasonal influenza-associated pediatric deaths reported by all state health departments	30	35	0
State and Territorial Epidemiologists Report	State-level assessments of influenza activity	Regional activity in 3 states (KY, NY, CT); focal activity in 4 states (HI, ME, MA, PA) and District of Columbia; sporadic or no activity in 48 states	Sporadic activity in 25 states; no Activity in 25 states	Local activity in 2 states (AL, HI); sporadic activity in 6 states (CA, ID, TX, FL, LA, NE); no activity in 43 states
National Respiratory and Enteric Virus Surveillance System	Predominant circulating virus	Influenza B	Influenza B	Influenza A
US WHO and Global Influenza Surveillance Network; National Respiratory and Enteric Virus Surveillance System (NREVSS)	Percentage of laboratory specimens positive and ILI positive for influenza virus	9.4	6.3	1.0

*CDC, Centers for Disease Control and Prevention; ILI, influenza-like illness as defined by CDC (*6*,*12*); WHO, World Health Organization. †Weeks 23, 30, 33, and 37 data were not available because CDC reported results only through week 20 in the 2005–06 influenza season and began reporting results on week 40 in the 2006–07 influenza season. The last day of the CDC week reports vary by several days from the last day of the survey results reported in Tables 3 and 4.

## Discussion

As shown in Table 4, our surveys of 7,268 adult respondents provided nationwide, prepandemic baseline information about household contact with cats (*10*) (28%–37%) and chickens (1%–4%), factors that may be relevant to the spread of currently circulating strains of avian influenza A (H5N1) virus. The data collected in 7 surveys over a 7-month period of low influenza activity were consistent with surveillance data gathered and reported by SPN.

More than 80% of persons in the populations and demographic subgroups surveyed nationwide agreed to participate in our study; this rate was similar for all regions of the country. The rate of self-reported illness from “flu” ranged consistently from 2% to 5% over the 7-month survey period, a time of low influenza activity and without reported human illnesses from highly pathogenic avian influenza A (H5N1) in the United States. Because only 30% (68/230) of respondents who self-reported the flu also self-reported fever, it follows that only a very small fraction of all respondents (0.9%, 68/7268) may have met CDC’s definition of ILI (i.e., fever [>100°F (37.8°C)] and cough or sore throat) (*12*). Similarly, during the same time frame, the SPN reported outpatient ILI rates of 1.2% and 0.9%, and the other CDC surveillance systems (Table 5) reported only regional and sporadic ILI in most states; moreover, none of the CDC-measured indicators suggested that influenza-related illness or death were excessive on the dates our surveys were conducted.

A comparison with CDC surveillance data suggests that the household telephone survey produced plausible, reproducible, and accurate results during a period when the circulation of influenza (predominantly influenza B virus) in the community was minimal. These results, when interpreted in the context of all other applicable surveillance reports, suggests that a direct telephone survey of adults at home could improve situational awareness of an influenza pandemic.Had unexpected trends or inconsistent rates of illness or apparent geographic disparities been identified, an analytic study could have been conducted to suggest possible risk factors or further investigations.

According to the DHHS Pandemic Influenza Plan, “Some states are considering the use of systematic phone surveys to supplement SPN data during a pandemic by providing estimates of local cases and affected households. CDC will explore the utility and feasibility of conducting this type of survey on a national level” (*2*). In a recent national telephone survey of 2,075 persons in Sweden, responses to the question “Did you have the ‘flu’ last week?” provided useful public health information regarding ongoing influenza disease activity (*13*). The survey took only 125 working hours to complete and cost approximately €3,250 (US $4,150). On the basis of our results, and assuming appropriate IRB approvals are already in place, we estimate that a new questionnaire could be developed in conjunction with public health officials and the telephone survey partners within 1 to 2 days, and the surveys could be conducted over the next 2 days with results delivered essentially immediately to public health officials. The direct cost of conducting each of the 2- to 3-day telephone surveys involving 1,200 adults and asking 10 questions was estimated to be $14,000; the 7-survey project in Tables 2–4 had a direct cost of ≈$98,000. In the United States, CDC recently adapted an ongoing national household telephone survey project, the Behavioral Risk Factor Surveillance System (BRFSS), to measure and monitor influenza vaccination coverage during the 2004–05 influenza season (*14*). CDC surveyed 26,526 adults during February 1–27, 2005 (*14*), and reported that ≈6,363 (24%) had been vaccinated. This CDC study also showed that influenza vaccination coverage among adults through January of the 2004–05 influenza season was greatest among persons >65 years of age (62.7%). In the telephone survey reported here, 21% (49/230) of those adults who self-reported the flu had received an annual influenza vaccine. The rates of influenza immunization reported in our August, September, and October 2006 surveys were lower than those in the early months of 2006, possibly because the 2006–07 trivalent immunizations were not yet available through healthcare providers at that time; however, this trend in coverage was similar to the monthly coverage trends reported by CDC’s BRFSS in 2004–05 (*14*).

Commercial polling agencies already elicit personal information from the public on an ongoing basis. Recently, as demonstrated by its adaptation of the BRFSS system, CDC was able to obtain information in response to a new public health problem by adding a few questions to an existing telephone survey. Although this adaptation of the survey, data analysis, and reporting infrastructure of the BRFSS appears to have been successful for determining influenza health status, it has apparently not yet been translated into a permanent surveillance system or ongoing capability. Moreover, the BRFSS might be overwhelmed in a pandemic emergency and, on very short notice, be forced to use additional telephone surveys that exist in the private and academic sectors. The adaptation of a private opinion survey company’s capabilities in partnership with an academic medical center through approved protocols could create a feasible, safe, inexpensive, flexible, and acceptable way of deriving public health information in emergencies and improving situational awareness.

Our study has several limitations. First, the telephone surveys were conducted only during months with low influenza activity in 2006. Second, although 7,268 adults were asked if they had “the flu,” only those who responded affirmatively (230 adults) were asked further questions about their influenza vaccination status and the presence of fever, cough, and sore throat. Biases may have been introduced based on the sequential approach to these questions, because some persons with fever, cough, or sore throat may have been misclassified by answering “No” to have the flu; additional follow-up questions about these ILI-related symptoms were not asked of all those surveyed. Third, this study had limitations similar to those of CDC’s BRFSS: 1) being a land-line telephone–based survey, our study excluded adults in households without telephones and adults who use only cellular telephones; and 2) because the data were self-reported and subject to recall bias, especially for questions that required recall over a longer period, frequency estimates might be less precise for some conditions or behaviors. Fourth, our estimates of the proportion of adults with nondirectory-listed telephone numbers including those who had ILI (*12*) were based on unmeasured self-reported temperatures (*13*) rather than on direct observations by healthcare providers, as in the SPN intended for ambulatory populations in healthcare facilities and clinics. Fifth, other illnesses and other viral infections besides influenza can cause ILI and can be accompanied by fever, cough, or sore throat. Finally, there was no laboratory confirmation of influenza or ILI in our survey participants.

Nonetheless, we were able to analyze our data in terms of self-reported fever, cough, or sore throat, thereby enabling us to estimate the proportion of respondents who met CDC’s definition of ILI (*12**,**13*). This capability might be useful during an actual pandemic, when it might be more desirable to assess the health status of patients by telephone (*15*) rather than exposing these and other patients and healthcare workers to the risk for healthcare-associated transmission of respiratory pathogens in clinical settings. Although the SPN’s nationwide estimate of ILI, determined using CDC criteria, was similar to our telephone survey-based estimate for the periods of low influenza activity in 2006, more data are needed to assess the performance of these methods during seasonal influenza peaks and during a pandemic. Others have suggested that sentinel surveillance studies typically underestimate influenza in a population; thus, telephone surveys may prove to be an increasingly important component of influenza surveillance (*13*).

## Conclusion

Telephone surveys might offer a practical solution to addressing the gaps in knowledge of influenza health status that might arise during a pandemic. Further telephone surveys should be performed during the peak influenza season to determine whether such an approach to surveillance would be a useful addition to ongoing influenza surveillance systems. The usefulness of the telephone survey to gain influenza immunization history and current ILI information on all people at risk should also be explored.
